# HEALTHCARE EXPERIENCES OF NHOPI AND OTHER U.S. ASIAN MEDICARE BENEFICIARIES

**DOI:** 10.1093/geroni/igz038.1603

**Published:** 2019-11-08

**Authors:** Megan K Beckett, Marc N Elliott, Katrin Hambarsoomian, Jacob Dembosky, Shondelle Wilson-Frederick, Sarah Gaillot, Steven Martino

**Affiliations:** 1 RAND Corporation, Santa Monica, California, United States; 2 RAND Corporation, Pittsburgh, Pennsylvania, United States; 3 Centers for Medicare & Medicaid Services, Baltimore, Maryland, United States

## Abstract

Native Hawaiians and Other Pacific Islanders (NHOPI) and Asians are the fastest-growing US racial and ethnic group. Differences in healthcare experiences may contribute to the better health outcomes of Asian-Only adults than NHOPIs, but little is known about NHOPI healthcare experiences. Using data from 1067 NHOPI and 9361 Asian-Only Medicare beneficiaries responding to the 2017 Medicare Consumer Assessment of Healthcare Providers and Systems (CAHPS) survey, we investigated NHOPI and Asian-Only differences in flu vaccination and six case-mix adjusted composite measures of healthcare experiences (scaled 0-100), with and without adjustment for Medicare coverage type and state of residence. Compared with Asian-Only beneficiaries, NHOPI beneficiaries reported better experiences getting needed care (adjusted-difference of +7 points), customer service (+5 points), doctor communication (+5 points), getting care quickly (+4 points), care coordination (+4 points), and getting needed prescription drugs (+3 points). In contrast, NHOPI beneficiaries reported worse flu immunization (-7 points; p<0.05 for all reported differences). Adjustment for Medicare Advantage (MA) coverage and state of residence accounted for <25% of differences. NHOPI vs. Asian-Only care experience differences were similar for MA and Fee-for-Service beneficiaries. NHOPI were immunized for influenza less often than Asian-Only beneficiaries in MA (-15 points), but not Fee-for-Service; this did not differ by gender. NHOPI men reported better experiences than Asian-Only men for customer service (+11 points) and getting care quickly (+6 points). Patient experience does not explain worse health outcomes found elsewhere for NHOPI than Asian-Only. Further study may suggest means of improving Asian-Only healthcare experiences and NHOPI immunization.

